# How Postal Currency Was Invented

**Published:** 1885-01

**Authors:** 


### HOW POSTAL CURRENCY WAS INVENTED.

Postal currency, which was the "change" during the war and until the resumption of specie payments, was the invention of Gen. Spinner, who had represented the Syracuse district of New York in Congress, and had been appointed Treasurer of the United States by President Lincoln. Small change had vanished, and in buying a dinner in the market change had to be taken in beets, cabbages, potatoes and what not. In his dilemma he bethought himself of the postage stamp. He sent down to the Post-office Department and purchased a quantity of stamps. He then ordered up a package of the paper upon which Government securities were printed. He cut the paper into various sizes. On the pieces he pasted stamps to represent different amounts. He thus initiated a substitute for fractional silver. This was not, however, a Government transaction in any sense; it could not be. Gen. Spinner distributed his improvised currency among the clerks of the department. They took it readily, and the trade folks more readily. The idea spread; the postage stamps, either detached or pasted upon a piece of paper, became the medium of small exchange. It was dubbed "postal currency." From this Gen. Spinner got his idea of the fractional currency, and went before Congress with it. That body readily adopted it, and but a short time after Gen. Spinner, had begun pasting operations a law was on the statute book providing for the issue of the fractional currency which became so popular. The facsimile of postage stamps was put on each piece of currency, and for a long time it was known as "postal currency." An enormous amount never was presented for redemption, and the Government was consequently the gainer.—*Ben. Perley Poore.*

